# Supplementary material for: Chemical multi-fingerprinting of exogenous ultrafine particles in human serum and pleural effusion
Source: Nat Commun. 2020 May 22;11:2567. doi: 10.1038/s41467-020-16427-x (PMC7244483; doi:10.1038/s41467-020-16427-x)
Supplement: Supplementary file 1 — Supplementary Information [file 41467_2020_16427_MOESM1_ESM.pdf]

SUPPLEMENTARY INFORMATION

for

Chemical multi-fingerprinting of exogenous ultrafine particles in human serum and pleural effusion

by Lu et al.

Contents

1. Supplementary experimental section
2. Supplementary Figures S1-S11
3. Supplementary Tables S1-S5
4. References for SI

1. Supplementary experimental section

Chemicals and reagents

Tetramethylammonium hydroxide (TMAH) was purchased from TCI (Shanghai) Development Co. (Shanghai, China). Benzonase was purchased from Xinhai Gene Testing Co. (Harbin, China). Proteinase K was purchased from Solarbio Co. (Beijing, China). The standard reference materials of silica nanoparticles (diameters: 50, 100, and 200 nm) were purchased from the NanoComposix (San Diego, CA, USA). Fe isotope standard reference material IRMM-634 was purchased from the Institute of Reference Materials and Measurement, (GEEL, Belgium). In-house standard (CAG-Fe) was kindly denoted by Prof. Xiangkun Zhu from the Chinese Academy of Geological Sciences (Beijing, China). The element calibration standard solution was purchased from Agilent (Santa Clara, CA, USA). Nitric acid was purchased from Merck (Darmstadt, Germany). Hydrochloric acid was from Beijing Chemicals Works (Beijing, China). Hydrogen peroxide was from Sinopharm Chemical Reagent Co. (Shanghai, China). Ultrapure water (18.3 MΩ·cm) produced from a Milli-Q Gradient system (Millipore, Bedford, USA) was used throughout the experiments. The sterile water was produced by a vertical high pressure steam sterilizer (Jiangyin Binjiang, Jiangsu, China).

Rough evaluation of inflammatory effect of the extracted particles

The pro-inflammatory risk of the particles extracted from human PE samples was evaluated using RAW 264.7 cells by analyzing the TNF-α release[1]. The RAW 264.7 murine cell line (Catalog No. TIB-71) was purchased from American Type Culture Collection (ATCC, Rockville, MD). The RAW 264.7 cells were cultured in Dulbecco's modified Eagle's medium (DMEM) at 37 °C in an incubator with 95% air and 5% CO_2. To eliminate interference, the particles (including exposure and control groups) were washed to neutrality (pH = 7) with ethyl alcohol and sterile water for several times. The endotoxin level of the particles was also determined with the Genscript Kit (Piscataway, NJ, USA) prior to exposure to the RAW 264.7 cells. To reflect the true pro-inflammatory activity of the particles in the human body, the

exposure concentration of the extracted particles to RAW 264.7 cells was adjusted to the same level as that in the human PE. The level of TNF-α was determined using the ELISA kit according to the manufacturer's instructions (R&D Systems). The concentrations of the TNF-α and endotoxin were calculated based on the absorption value on a microplate reader (Varioskan Flash, Thermo, USA).

2. Supplementary figures

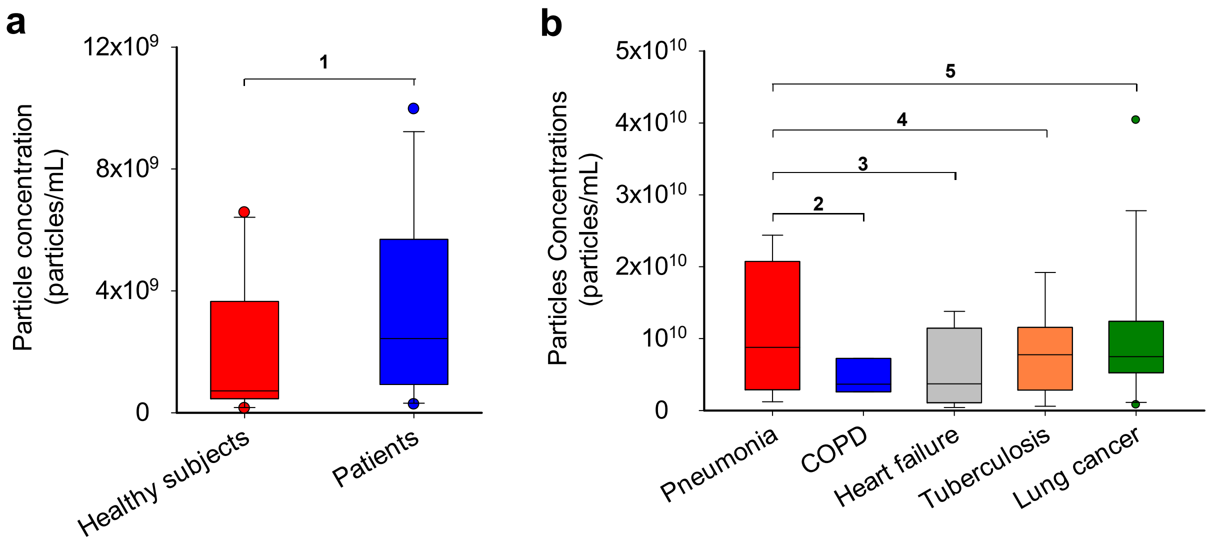

Supplementary Figure 1. Comparison of NP concentrations in the body fluids between healthy subjects and patients. (**a**) Comparison of NP concentrations in serum between healthy subjects and patients. $P_1 = 0.11$ (unpaired Student's two-tailed *t*-test). (**b**) Comparison of NP concentrations in PE among different diseases. $P_2 = 0.29$, $P_3 = 0.39$, $P_4 = 0.63$, and $P_5 = 0.95$ (unpaired Student's two-tailed *t*-test). In the box plots, bounds of the box spans from 25% to 75% percentile, center line represents median, and whiskers visualize 5% and 95% of the data points. Source data are provided as a Source Data file. From **a**, it can be seen that NPs in the serum of patients show a wider concentration range than that of healthy subjects. Despite that, no significant difference in NPs concentrations are observed between healthy subjects and patients ($P > 0.1$, unpaired Student's two-tailed *t*-test), suggesting that the NPs are widely presented in human body regardless of diseases. From **b**, no significant differences are observed among different diseases ($P > 0.2$, unpaired Student's two-tailed *t*-test), indicating that the enrichment of NPs in PE is not caused by a specific disease. As long as PE is caused, it can be a potential reservoir for NPs in the human body.

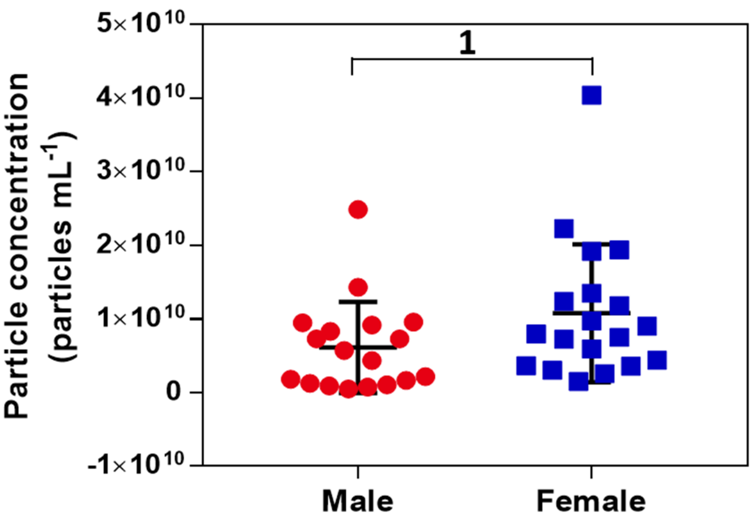

Supplementary Figure 2. Comparison of particle concentrations in PE samples between different sexes. $P_1 = 0.11$ (unpaired Student's two-tailed *t*-test). The NP concentration in PE shows no significant correlation with sex. Error bars represent the mean ± s.d. of the particle concentrations ($n = 18$ or 19). Source data are provided as a Source Data file.

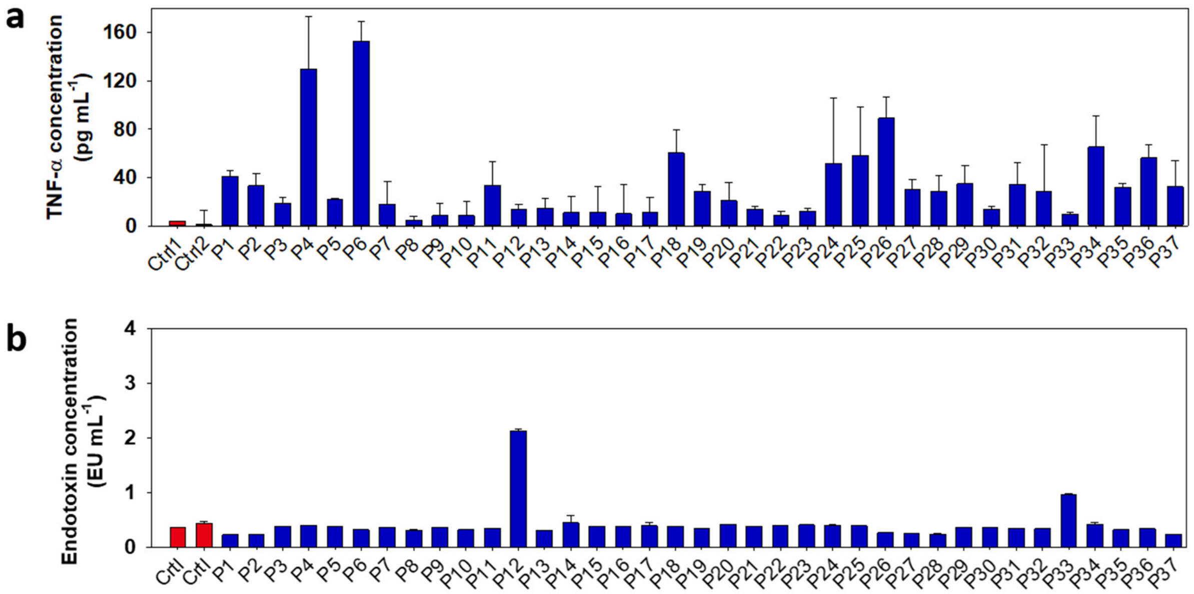

Supplementary Figure 3. Rough evaluation of the pro-inflammatory effects of the PE-derived particles. (**a**) TNF-α concentration after exposure of the PE-derived particles to RAW 264.7 cells, (**b**) endotoxin levels of the PE-derived particles. The error bars represent 1SD from the parallel experiments (n = 3). For comparison, the results for two blank control samples are also presented. Source data are provided as a Source Data file. It can be seen from **a** that the PE-derived NPs could cause pro-inflammatory effects for all PE samples, suggesting that the NPs inhaled might have potential health risks. From **b**, except for only P12 and P33 samples, all extracted NPs showed similarly low levels of endotoxin as that of control, suggesting that the endotoxin levels could be negligible in these samples and the inflammatory effect of the extracted NPs should be caused by the particles themselves. It should be emphasized that this was a very preliminary test for the toxicological effect of the PE-derived NPs. It might not necessarily reflect the real toxicity of the particles in the human body because only a single cell type and a single dose was used. Due to the NP extraction process used in the present study, the surface properties (e.g., surface chemistry, redox potential, and solubility) of NPs might have been altered compared with those in the human body, which may greatly affect the toxicity of the NPs. Thus, a more comprehensive study is needed to elucidate the toxicological effects of the NPs.

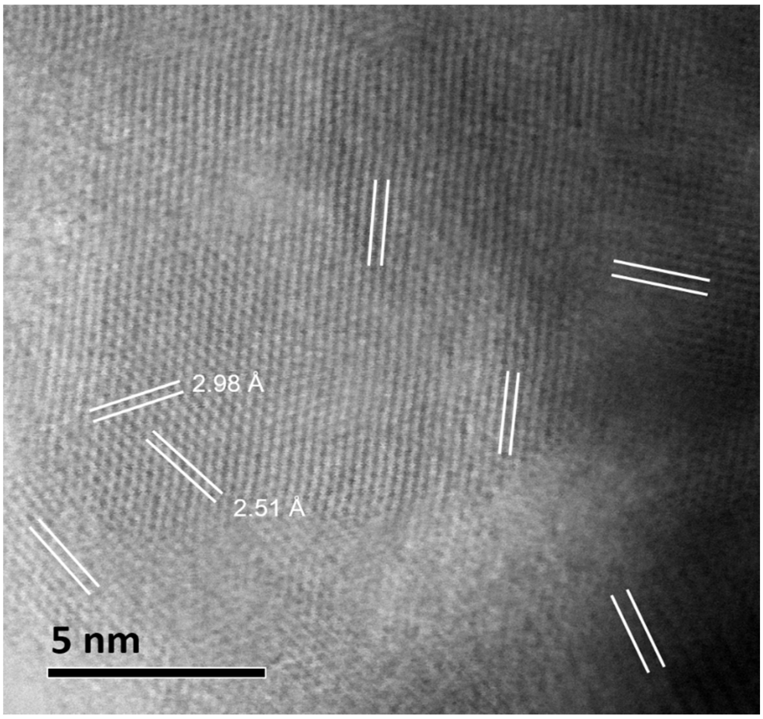

Supplementary Figure 4. High-resolution HAADF-STEM image of a particle shown in Fig. 2e. Indexing of the lattice fringes is consistent with the magnetite crystal structure. Furthermore, the particle display fused interlocking surface crystallites (noting the varying orientations of the individual crystallite faces), typical of high-temperature (e.g., combustion) formation and then crystallization upon rapid cooling and/or oxidation[2,3], which bear high similarity with magnetite NPs abundantly present in ambient PM[2].

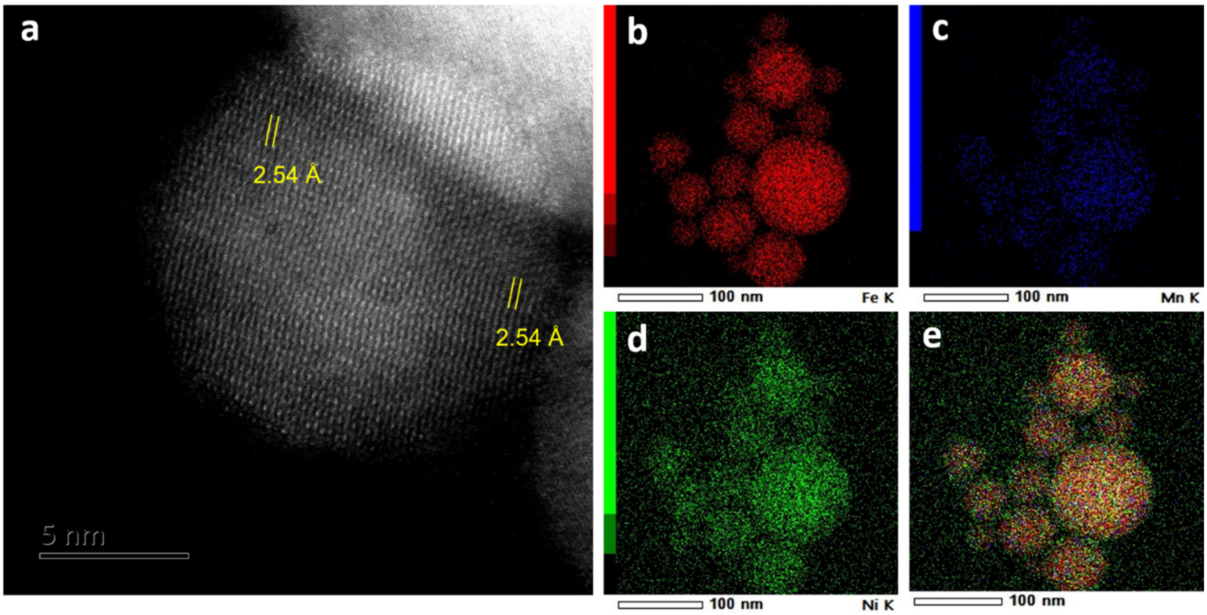

Supplementary Figure 5. Details of particles shown in Fig. 2h. (**a**) High-resolution HAADF-STEM image of a rounded particle marked by the yellow arrow in Fig. 2h. **(b-e)** EDXS mapping of the particles shown in Fig. 2h. It is shown that the particles have a crystal structure and only consist of Fe, Mn, and Ni. These elements are frequently contained in fly ash released from coal combustion. However, no Si or Al which are common components of fly ash are found. Thus, it is more likely that these particles are Fe-Mn-Ni alloy particles existing in PM emitted from ferroalloy plants[4,5].

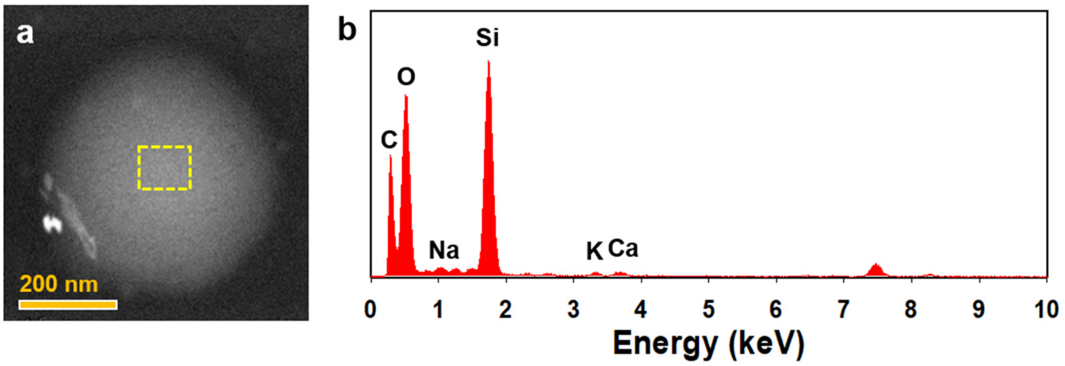

Supplementary Figure 6. EDXS characterization of the spherical particles shown in Fig. 2n. (**a**), HAADF-STEM image of a typical particle. (**b**) The corresponding EDXS spectrum of the highlighted section in **a**. Source data are provided as a Source Data file. This particle shows a relatively large size (diameter ~400 nm) and does not show any crystal structure. From the EDXS analysis, it mainly consists of C, O, and high abundance of Si. The spherical amorphous Si-containing particles highly match with the characteristics of fly ashes, a frequent content in airborne particulate matter[6-8]. Furthermore, it is noteworthy that the particle size is significantly larger that the commonly supposed threshold for ambient UFPs (< 0.1 μm) to penetrate through the pulmonary alveoli. On one hand, the similar phenomenon was also observed in animals that large particles (e.g., 240 nm) could translocate into blood via inhalation pathway[9]. On the other hand, the potential agglomeration tendency of NPs in body fluids and in extraction process may also cause large variation in particle size. Thus, further studies are still needed to determine whether a large particle like this really likely to translocate via inhalation.

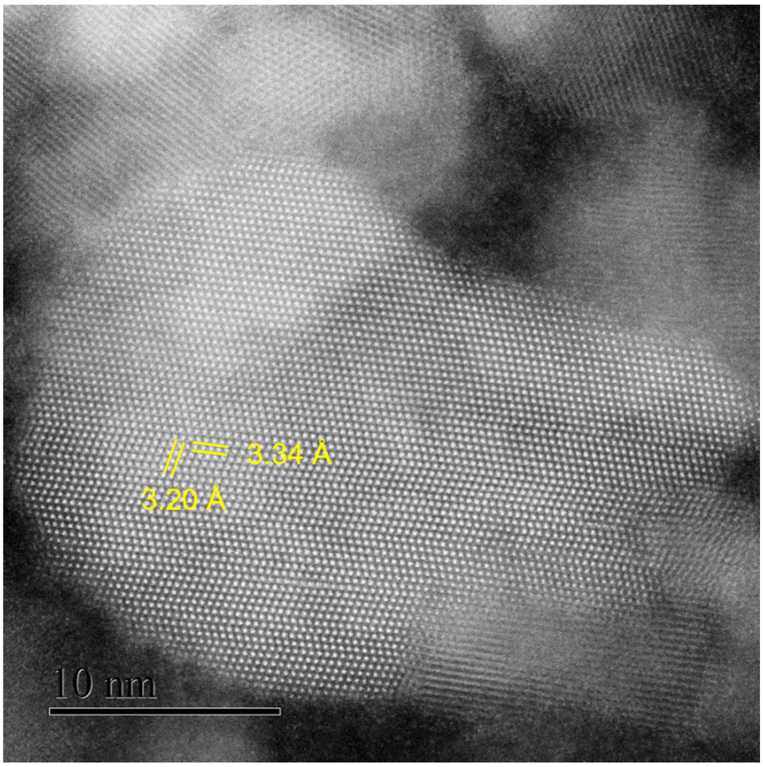

Supplementary Figure 7. High-resolution HAADF-STEM image of particles shown in Fig. 2v. Indexing of the lattice fringes is consistent with HgS. This result is also verified by the EDXS element mapping and SAED pattern (see Fig. 2w-x). Hg and its compounds are toxic substances, and Hg in PM is normally thought to come from coal burning. It should be noted that, although we have found HgS particles by STEM, the Hg-bearing particles in the body fluids are actually very rare so that no Hg signals can be detected by ICP-MS (see Fig. 1f).

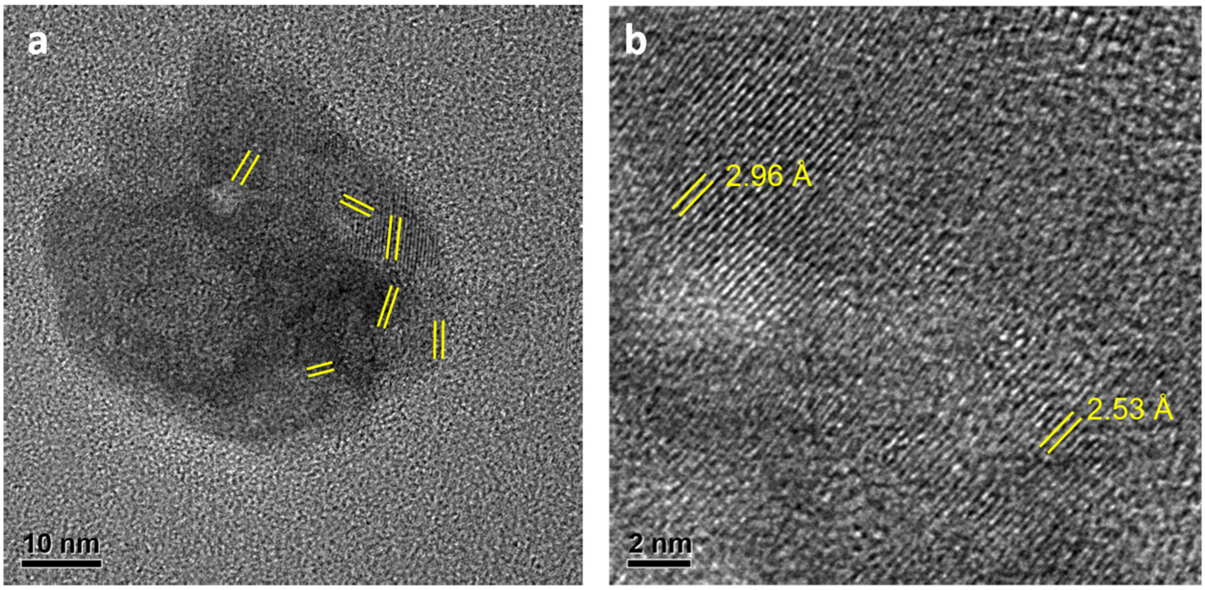

Supplementary Figure 8. High-resolution HAADF-STEM image of particles shown in Fig. 3a. (**a**) HAADF-STEM image of a particle (~50 nm) marked in the cyan square in Fig. 3a. (**b**) High-resolution HAADF-STEM image of the particle showing the lattice fringes that are fully consistent with the magnetite crystal structure. Note the particle also bear fused interlocking surface crystallites with the varying orientations of the individual crystallite faces (**a**), typical of high-temperature sources (e.g., combustion)[2,3], as magnetite NPs found in PE (see Fig. 2e and Supplementary Fig. 4). Therefore, it is suggested that the magnetite NPs found in PE and serum should be of same origin from ambient PM.

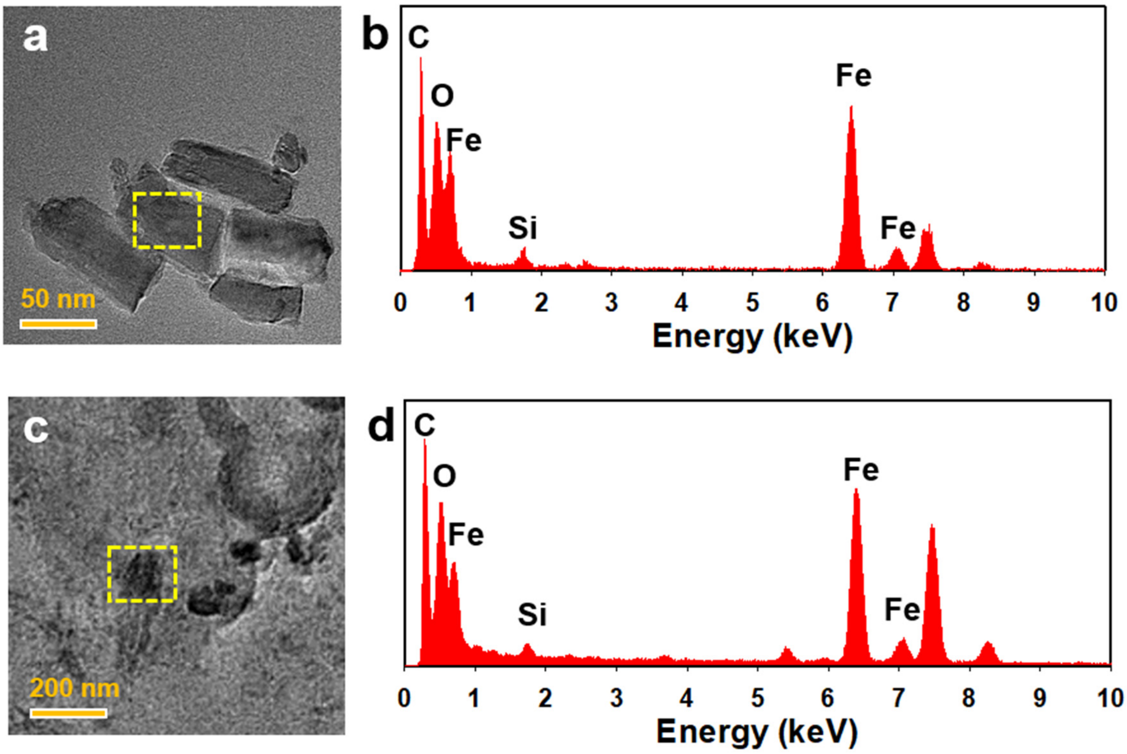

Supplementary Figure 9. Characterization of some Fe-rich NPs extracted from human serum samples. (**a**,**c**) NPs extracted two different subjects. (**b**,**d**) The corresponding EDXS spectra of the sections marked in yellow squares in **a** and **c**. Source data for **b** and **d** are provided as a Source Data file. The particles in **a** and **c** have quite similar elemental compositions mainly consisting of Fe, O, Si, etc., but they show totally different shapes. The particles in **a** have a euhedral angular crystal morphology, while those in **c** show blurry boundaries. Their morphologies and elemental compositions are also different from the rounded magnetite crystal NPs shown in Fig. 2b-g. These results indicate the high complexity of Fe-bearing particles in the human body. Although the sources of these Fe-rich particles are still not clear, their sizes (up to ~150 nm) appear to be much larger than endogenous magnetite particles formed via in situ crystallization within the 8-nm-diameter core of ferritin. Thus, we infer that they might be of external origins.

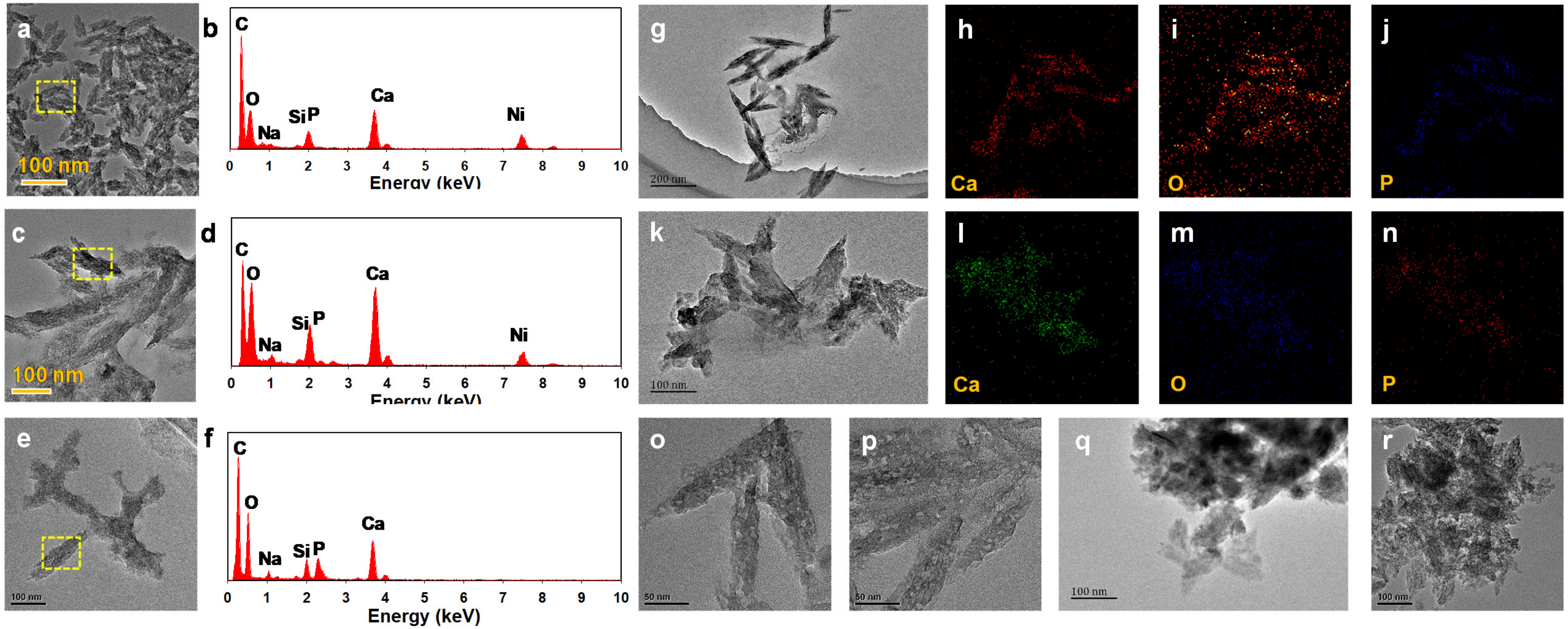

Supplementary Figure 10. Spindle-shaped NPs found in both serum and PE samples. (**a-b**, **e-f**, **g-j**, and **q-r**) NPs extracted from PE samples (P12, P15, P21, and P30). (**c-d**, **k-n**, and **o-p**) NPs extracted from human serum samples (S32, S19, and S21). (**b**, **d**, and **f**) The corresponding EDXS spectra of the sections marked in yellow squares in **a**, **c**, and **e**. (**h-j** and **l-n**) EDXS mapping of the particles shown in **g** and **k**. Source data for **b**, **d** and **f** are provided as a Source Data file. We can observe a large number of spindle-shaped particles with a mineral-like elemental composition (C, O, Ca, and P) in human PE samples (**a**, **e**, **g**, **q**, and **r**). Interestingly, we have also identified the presence of the particles with a similar morphology in human serums (**c**, **k**, **o**, and **p**). These spindle-shaped particles in serum and PE samples have nearly identical elemental fingerprints (**b**, **d**, **f**, **h-j**, and **l-n**). Considering together their quite similar morphologies, it is rational to infer that they are from the same sources. The elemental composition (C, O, Ca, and P) may be suggestive of calcium carbonate and calcium phosphate which have both exogenic or endogenic origins. However, the currently available information is not sufficient to determine whether these particles are of external source or formed internally.

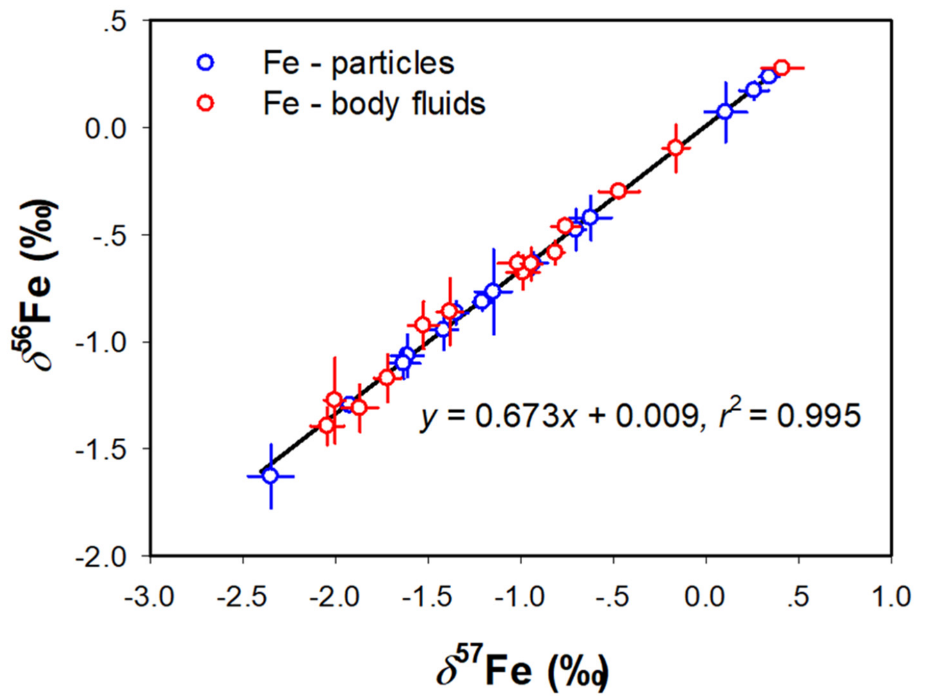

Supplementary Figure 11. Three-isotope plot showing δ^{56}Fe *versus* δ^{57}Fe for all tested samples. The black line represents the mass-dependent fractionation line. The error bars represent 1s.d. from parallel measurements (n = 3). It can be seen that no mass-independent fractionation is observed with the tested samples. Source data are provided as a Source Data file.

3. Supplementary tables

Supplementary Table 1. Summary of the study participants.[a]

Characteristics	Healthy subjects (n = 19)	All patients (n = 37)	Benign (n = 19)	Malignant (n = 18)
Gender				
Male (%)	7 (36.8)	18 (48.6)	10 (52.6)	8 (44.4)
Female (%)	12 (63.2)	19 (51.4)	9 (47.4)	10 (55.6)
Age (years)				
Mean ± SD	38 ± 11	63 ± 17	60 ± 20	69 ± 12
Histology				
Non-small cell lung adenocarcinoma	-	15	-	-
Small cell lung adenocarcinoma	-	1	-	-
Breast cancer	-	2	-	-
Non-malignant	-	19	-	-
Subtypes for non-malignant cases				
Pneumonia	-	-	4	-
Tuberculosis	-	-	6	-
COPD	-	-	4	-
Heart failure	-	-	4	-
Other	-	-	1	-

[a] The serum samples used in this study were collected from both healthy subjects (n = 19) and patients with benign diseases (n = 18; see Supplementary Table 2), and the PE samples were all collected from patients with different diseases (n = 37; see Supplementary Table 3).

Supplementary Table 2. Information of participants whose serum samples were collected.[a]

No.	Gender	Age (year)	Disease
1	M	32	Healthy
2	M	36	Healthy
3	M	42	Healthy
4	M	27	Healthy
5	M	57	Healthy
6	M	26	Healthy
7	M	34	Healthy
8	F	23	Healthy
9	F	50	Healthy
10	F	55	Healthy
11	F	56	Healthy
12	F	53	Healthy
13	F	24	Healthy
14	F	29	Healthy
15	F	32	Healthy
16	F	43	Healthy
17	F	39	Healthy
18	F	27	Healthy
19	F	40	Healthy
20	M	67	COPD[a]
21	M	84	COPD
22	M	77	Pneumonia
23	M	73	COPD
24	M	92	Heart failure
25	M	66	Heart failure
26	M	21	Tuberculous pleuritis
27	M	72	Pneumonia
28	M	76	Tuberculosis & COPD
29	F	53	Pneumonia
30	F	62	COPD
31	F	81	Heart failure
32	F	68	Heart failure
33	F	67	POEMS syndrome
34	F	30	Tuberculous pleuritis
35	F	25	Pneumonia
36	F	51	Tuberculous pleuritis
37	F	53	Tuberculous pleuritis

[a] COPD: chronic obstructive pulmonary disease.

Supplementary Table 3. Information of the patients whose PE samples were collected.

No.	Gender	Age (year)	Disease
1	F	67	POEMS syndrome
2	F	83	Lung cancer
3	F	46	Lung cancer
4	F	68	Lung cancer
5	F	54	Lung cancer
6	M	84	COPD
7	F	81	Heart failure
8	M	77	Pneumonia
9	M	73	COPD
10	F	25	Pneumonia
11	M	85	Lung cancer
12	F	68	Heart failure
13	F	55	Lung cancer
14	F	71	Breast cancer
15	M	75	Lung cancer
16	M	83	Lung cancer
17	M	92	Heart failure
18	F	87	Lung cancer
19	F	69	Lung cancer
20	F	30	Tuberculous pleuritis
21	M	66	Heart failure
22	M	21	Tuberculous pleuritis
23	F	65	Lung cancer
24	F	62	COPD
25	M	72	Pneumonia
26	M	75	Lung cancer & COPD
27	M	69	Lung cancer
28	M	76	Tuberculosis & COPD
29	F	53	Pneumonia
30	F	52	Breast cancer
31	F	51	Tuberculous pleuritis
32	M	67	COPD
33	M	58	Lung cancer
34	M	34	Tuberculous pleuritis
35	M	61	Lung cancer
36	M	80	Lung cancer
37	F	53	Tuberculous pleuritis

Supplementary Table 4. Elemental concentrations of the particles extracted from human PE samples (ng mL^{-1}).

ID	Al	V	Cr	Mn	Fe	Co	Ni	Cu	Zn	As	Se	Sr	Ag	Cd	Ba	U	Ti	Pt
P01	175±34	0.14±0.06	0.69±0.20	5.7±0.1	14±2	0.06±0.06	ND	ND	ND	ND	ND	ND	ND	ND	4.8±0.2	ND	20±1	0.02±0.03
P02	278±10	0.11±0.07	0.49±0.21	5.0±0.1	39±4	ND	ND	ND	1.1±0.2	ND	ND	ND	ND	ND	1.9±0.5	ND	12±1	ND
P03	163±20	0.38±0.07	0.94±0.22	10±1	25±4	ND	ND	ND	ND	ND	ND	2.6±0.2	0.37±0.03	ND	1.2±0.4	ND	11±1	ND
P04	330±16	0.46±0.11	0.51±0.16	4.5±0.1	17±1	0.06±0.04	3.6±0.4	43±1	21±1	ND	ND	13±1	ND	ND	4.2±0.6	ND	5.8±0.2	ND
P05	56±14	0.63±0.07	0.20±0.06	3.8±0.1	22±2	ND	ND	ND	ND	ND	ND	4.2±0.2	ND	ND	1.9±0.2	ND	ND	ND
P06	ND	0.75±0.15	2.4±0.2	2.9±0.1	24±3	0.11±0.02	ND	ND	ND	ND	ND	15±1	ND	ND	7.5±0.8	ND	ND	ND
P07	169±25	0.82±0.08	1.3±0.1	1.8±0.1	22±2	ND	2.7±0.7	0.21±0.05	ND	ND	ND	3.8±0.1	ND	ND	4.4±0.4	ND	15±1	ND
P08	135±16	0.59±0.10	0.98±0.13	21±1	46±4	ND	ND	ND	3.5±0.7	ND	ND	11±1	ND	ND	7.2±0.5	ND	ND	0.04±0.02
P09	ND	ND	0.33±0.09	44±1	4.6±0.8	ND	ND	ND	1.3±0.3	ND	ND	ND	ND	ND	5.2±0.6	ND	ND	ND
P10	84±2	0.41±0.08	0.80±0.05	13±1	16±2	0.06±0.02	ND	ND	ND	ND	1.2±0.2	ND	0.35±0.05	ND	2.3±0.4	ND	ND	ND
P11	120±17	0.56±0.11	ND	15±1	4.3±0.5	0.14±0.04	ND	0.17±0.51	ND	ND	ND	ND	ND	ND	6.2±0.5	ND	5.0±0.3	ND
P12	ND	0.56±0.10	ND	54±1	48±3	0.07±0.03	ND	ND	ND	ND	ND	ND	ND	ND	7.0±0.6	ND	ND	0.02±0.08
P13	1140±25	ND	0.23±0.04	1.5±0.1	3.9±0.5	ND	ND	ND	ND	ND	ND	ND	ND	ND	ND	ND	ND	ND
P14	109±8	0.28±0.09	ND	ND	7.1±0.5	ND	ND	ND	ND	ND	1.6±0.3	ND	ND	ND	ND	ND	16±1	ND
P15	105±5	0.71±0.08	0.16±0.08	4.6±0.1	4.2±0.8	ND	ND	0.20±0.09	0.42±0.18	ND	4.2±0.7	ND	ND	ND	4.6±0.7	ND	17±1	ND
P16	398±35	ND	0.64±0.13	41±1	5.5±0.1	ND	3.3±0.2	0.17±0.11	3.7±0.2	ND	ND	5.6±0.2	ND	ND	7.2±0.6	ND	ND	ND
P17	ND	0.38±0.11	0.29±0.08	ND	2.3±0.8	ND	ND	ND	ND	ND	ND	ND	ND	ND	ND	ND	6.9±0.8	ND
P18	105±6	ND	0.21±0.04	ND	ND	ND	ND	0.51±0.17	ND	ND	ND	ND	ND	ND	ND	ND	ND	ND
P19	ND	0.30±0.07	0.38±0.09	14.3±0.1	7.7±1.0	ND	ND	ND	2.2±0.3	ND	ND	18±1	ND	ND	4.2±0.9	ND	6.5±0.8	ND
P20	75±16	0.25±0.06	ND	ND	4.3±0.3	ND	ND	ND	ND	ND	ND	ND	ND	ND	ND	ND	ND	ND
P21	83±15	0.82±0.12	ND	45.7±0.1	5.0±0.8	ND	1.2±0.1	ND	3.2±0.1	ND	ND	45±1	ND	ND	5.6±0.7	ND	ND	ND
P22	795±43	0.63±0.09	0.47±0.19	ND	24±1	ND	ND	ND	ND	ND	ND	ND	ND	ND	ND	ND	ND	ND
P23	68±13	0.32±0.10	1.7±0.2	ND	63±5	ND	2.6±0.7	ND	ND	ND	ND	ND	ND	ND	ND	ND	ND	ND
P24	154±12	0.57±0.08	0.49±0.07	17.3±0.1	76±2	0.11±0.02	ND	ND	1.9±0.2	ND	1.67±0.29	13±1	0.37±0.03	ND	3.7±0.7	ND	ND	ND
P25	105±27	0.13±0.09	0.99±0.18	ND	7.0±1.6	ND	ND	ND	ND	ND	ND	ND	ND	ND	ND	ND	ND	ND
P26	225±35	0.80±0.12	ND	ND	6.6±0.7	ND	ND	ND	ND	ND	ND	ND	ND	ND	ND	ND	ND	ND

P27	79±15	0.58±0.11	0.43±0.07	ND	30±1	ND	ND	39.9±2.9	3.1±0.5	ND	ND	ND	0.49±0.08	ND	ND	ND	ND	ND
P28	109±24	0.26±0.05	ND	ND	4.5±0.3	ND	ND	ND	ND	ND	ND	ND	ND	ND	0.75±0.17	ND	ND	ND
P29	98±28	0.36±0.00	1.5±0.2	ND	3.8±0.2	ND	ND	ND	ND	ND	ND	ND	0.30±0.04	ND	ND	ND	ND	ND
P30	86±13	0.65±0.13	0.48±0.04	ND	4.2±1.3	ND	ND	ND	ND	ND	ND	ND	ND	ND	ND	ND	ND	ND
P31	488±16	1.8±0.1	0.68±0.08	0.3±0.1	6.8±1.2	0.06±0.01	0.7±0.2	ND	ND	ND	ND	ND	0.28±0.01	ND	ND	ND	30±1	ND
P32	109±15	0.51±0.10	0.28±0.06	ND	87±4	ND	ND	ND	ND	ND	ND	ND	ND	ND	ND	ND	ND	ND
P33	570±32	2.5±0.2	0.76±0.12	0.3±0.1	8.4±1.0	0.06±0.02	ND	ND	ND	ND	ND	ND	0.34±0.07	ND	ND	ND	37±1	ND
P34	233±28	1.2±0.1	0.35±0.09	ND	2.4±0.5	ND	ND	ND	ND	ND	ND	ND	ND	ND	ND	ND	4.4±0.9	ND
P35	311±31	0.14±0.07	ND	ND	5.1±0.6	ND	ND	ND	ND	ND	ND	ND	ND	ND	ND	ND	14±1	ND
P36	641±43	2.3±0.2	1.1±0.2	1.2±0.1	4.4±0.6	0.08±0.01	0.4±0.1	0.32±0.26	5.3±0.3	ND	ND	ND	ND	ND	2.3±0.3	ND	40±1	ND
P37	131±34	0.69±0.09	ND	ND	3.1±0.5	ND	ND	ND	1.1±0.3	ND	1.47±0.26	ND	ND	ND	ND	ND	ND	ND

ND: not detected.

Supplementary Table 5. Parameters for Fe isotope ratio measurement by MC-ICP-MS.

Sample preparation	
Digestion	Acid assisted, 110 °C, 14 h
Column purification	Strong anion exchange resin (AG MP-1), 100-200 mesh
Ni doping for Fe (yes/no)	no
Instrument settings	
Sample introduction for Fe	Dry plasma: DeSolvation Nebulizer (DSN-100)
	PFA nebulizer, uptake rate ~70 µL/min
Sampler cone (nickel)	"experimental" WA cone (Nu Instruments)
Skimmer cone (nickel)	"experimental" WA cone (Nu Instruments)
Lens settings	Optimized for maximum analytical signal intensity
Torch	Glass
Collector	L5 – ^{54}Fe, Ax – ^{56}Fe, H3 – ^{57}Fe
RF power	1300 W
Data acquisition parameters	
Scan type	Static
Measurement mode	Medium-resolution (Resolution power ~ 7600) for Fe
	Standard-sample-standard bracketing
Measurement intensity (^{56}Fe)	6 V/ppm for ^{56}Fe
Blank signal (3%) HNO_3 in ^{56}Fe	< 20 mV
Magnet delay time	2 s
Number of blocks	3 block, 10 cycles
Integration time	10 s

4. References for SI

1 Qu, G. *et al.* Improved Biocompatibility of Black Phosphorus Nanosheets by Chemical Modification. *Angew. Chem. Int. Ed.* **56**, 14488-14493 (2017).

2 Maher, B. A. *et al.* Magnetite pollution nanoparticles in the human brain. *P. Ntl. Acad. Sci. USA* **113**, 10797-10801 (2016).

3 Calderon-Garciduenas, L. *et al.* Combustion- and friction-derived magnetic air pollution nanoparticles in human hearts. *Environ. Res.* **176**, 108567 (2019).

4 Romanski, A. & Konstanty, J. Ball-milled Fe-Ni and Fe-Mn matrix powders for sintered diamond tools. *Arch. Metall. Mater.* **59**, 189-193 (2014).

5 Marris, H., Deboudt, K., Flament, P., Grobety, B. & Giere, R. Fe and Mn oxidation states by TEM-EELS in fine-particle emissions from a Fe-Mn alloy making plant. *Environ. Sci. Technol.* **47**, 10832-10840 (2013).

6 Senlin, L. *et al.* The relationship between physicochemical characterization and the potential toxicity of fine particulates ($PM_{2.5}$) in Shanghai atmosphere. *Atmos. Environ.* **42**, 7205-7214 (2008).

7 Silva, L. F. *et al.* The occurrence of hazardous volatile elements and nanoparticles in Bulgarian coal fly ashes and the effect on human health exposure. *Sci. Total Environ.* **416**, 513-526 (2012).

8 Li, H., Zhang, J., Zhao, Y., Wu, C.-Y. & Zheng, C. Wettability of fly ashes from four coal-fired power plants in China. *Ind. Eng. Chem. Res.* **50**, 7763-7771 (2011).

9 Kato, T. *et al.* Evidence that exogenous substances can be phagocytized by alveolar epithelial cells and transported into blood capillaries. *Cell Tissue Res.* **311**, 47-51 (2003).
