# Supplementary material for: Chemical multi-fingerprinting of exogenous ultrafine particles in human serum and pleural effusion
Source: Nat Commun. 2020 May 22;11:2567. doi: 10.1038/s41467-020-16427-x (PMC7244483; doi:10.1038/s41467-020-16427-x)
Supplement: Supplementary file 4 — Reporting Summary [file 41467_2020_16427_MOESM4_ESM.pdf]

nature research

Corresponding author(s): Qian Liu
Guibin Jiang

Last updated by author(s): Mar 7, 2020

Reporting Summary

Nature Research wishes to improve the reproducibility of the work that we publish. This form provides structure for consistency and transparency in reporting. For further information on Nature Research policies, see Authors & Referees and the Editorial Policy Checklist .

Statistics

For all statistical analyses, confirm that the following items are present in the figure legend, table legend, main text, or Methods section.

n/a	Confirmed	
☐	☒	The exact sample size (*n*) for each experimental group/condition, given as a discrete number and unit of measurement
☐	☒	A statement on whether measurements were taken from distinct samples or whether the same sample was measured repeatedly
☐	☒	The statistical test(s) used AND whether they are one- or two-sided<br>*Only common tests should be described solely by name; describe more complex techniques in the Methods section.*
☐	☒	A description of all covariates tested
☐	☒	A description of any assumptions or corrections, such as tests of normality and adjustment for multiple comparisons
☐	☒	A full description of the statistical parameters including central tendency (e.g. means) or other basic estimates (e.g. regression coefficient) AND variation (e.g. standard deviation) or associated estimates of uncertainty (e.g. confidence intervals)
☐	☒	For null hypothesis testing, the test statistic (e.g. *F*, *t*, *r*) with confidence intervals, effect sizes, degrees of freedom and *P* value noted<br>*Give P values as exact values whenever suitable.*
☐	☒	For Bayesian analysis, information on the choice of priors and Markov chain Monte Carlo settings
☐	☒	For hierarchical and complex designs, identification of the appropriate level for tests and full reporting of outcomes
☐	☒	Estimates of effect sizes (e.g. Cohen's *d*, Pearson's *r*), indicating how they were calculated

Our web collection on statistics for biologists contains articles on many of the points above.

Software and code

Policy information about availability of computer code

Data collection	No software was used.
Data analysis	OriginPro 2015 and SigmaPlot 12.5

For manuscripts utilizing custom algorithms or software that are central to the research but not yet described in published literature, software must be made available to editors/reviewers. We strongly encourage code deposition in a community repository (e.g. GitHub). See the Nature Research guidelines for submitting code & software for further information.

Data

Policy information about availability of data

All manuscripts must include a data availability statement. This statement should provide the following information, where applicable:

- Accession codes, unique identifiers, or web links for publicly available datasets
- A list of figures that have associated raw data
- A description of any restrictions on data availability

The source data underlying Figs 1a-f and 4 and Supplementary Figs S1, S2, S3, and S11 are provided as a Source Data file.

Field-specific reporting

Please select the one below that is the best fit for your research. If you are not sure, read the appropriate sections before making your selection.

☐ Life sciences ☐ Behavioural & social sciences ☒ Ecological, evolutionary & environmental sciences

For a reference copy of the document with all sections, see nature.com/documents/nr-reporting-summary-flat.pdf

Ecological, evolutionary & environmental sciences study design

All studies must disclose on these points even when the disclosure is negative.

Study description	Discovery and characterization of exogenous nanoparticles (NPs) in human serum and pleural effusion samples collected from non-occupational subjects by chemical multi-fingerprinting techniques (including elemental fingerprints, high-resolution structural fingerprints, and stable iron isotopic fingerprints).
Research sample	A group of human serum samples and a group of human pleural effusion (PE) samples collected from non-occupational subjects. The samples are meant to represent non-occupational population. The NPs in serum can reflect the internal exposure of ambient ultrafine particles (UFPs), and the PE was used as a host of UFPs in the human body. The information for all study participants are given in Supplementary Table 1-3.
Sampling strategy	The sample size was kept the same for serum and PE samples (n = 37). The PE samples were collected from 37 patients with different diseases because PE can only be clinically collected from patients with some specific diseases. The serum samples included 19 healthy individuals and 18 patients collected in the same region to make a comparison between healthy subjects and patients. The information for all study participants are given in Supplementary Table 1-3.
Data collection	Dawei Lu collected the data using the instruments noted in the Method section.
Timing and spatial scale	All samples were collected in the Pearl River Delta region, China.
Data exclusions	No data were excluded.
Reproducibility	All attempts to repeat the experiments were successful.
Randomization	The healthy individuals presented no clinical evidence of diseases, and the patients were classified by clinical diagnosis.
Blinding	Not relevant to this study.

Did the study involve field work? ☐ Yes ☒ No

Reporting for specific materials, systems and methods

We require information from authors about some types of materials, experimental systems and methods used in many studies. Here, indicate whether each material, system or method listed is relevant to your study. If you are not sure if a list item applies to your research, read the appropriate section before selecting a response.

Materials & experimental systems

n/a	Involved in the study	
☒	☐	Antibodies
☒	☐	Eukaryotic cell lines
☒	☐	Palaeontology
☒	☐	Animals and other organisms
☐	☒	Human research participants
☒	☐	Clinical data

Methods

n/a	Involved in the study	
☒	☐	ChIP-seq
☒	☐	Flow cytometry
☒	☐	MRI-based neuroimaging

Human research participants

Policy information about studies involving human research participants

Population characteristics	The information for all study participants are given in Supplementary Table 1-3. All participants who represented non-occupational population denied any history of occupational exposure to hazardous materials.
Recruitment	For PE samples, 37 patients who had PE and needed thoracentesis were recruited. For serum samples, 19 healthy individuals and 18 patients with lung diseases were recruited to make a comparison between healthy subjects and patients.
Ethics oversight	The study protocol were approved by the Ethics Committee of Shenzhen Institutes of Advanced Technology of Chinese Academy of Sciences.

Note that full information on the approval of the study protocol must also be provided in the manuscript.
